# Supplementary material for: Aseptic Abscess Syndrome: Clinical Characteristics, Associated Diseases, and up to 30 Years’ Evolution Data on a 71-Patient Series
Source: J Clin Med. 2022 Jun 25;11(13):3669. doi: 10.3390/jcm11133669 (PMC9267245; doi:10.3390/jcm11133669)
Supplement: Supplementary file 1 [file jcm-11-03669-s001.zip › jcm-1773633-supplementary.pdf]

Table S1. Treatments received by the 71 patients with an aseptic abscess syndrome

	**Total (n = 71)**
Corticosteroids, *n* (%)	61 (85.9)
Colchicine, *n* (%)	27 (38.0)
Immunosuppressants or immunomodulators, n (%)	44 (61.9)
Azathioprine	22 (30.9)
Cyclophosphamide	8 (11.2)
Methotrexate	3 (4.2)
Thalidomide	2 (2.8)
Dapsone	2 (4.2)
Leflunomide	1 (1.4)
Sulfasalazine	1 (1.4)
Ciclosporine	1 (1.4)
Tacrolimus	1 (1.4)
Mycophenolate mofetil	1 (1.4)
Biologics, n (%)	23 (32.3)
Infliximab	14 (19.7)
Adalimumab	8 (11.2)
Anakinra	4 (5.6)
Ustekinumab	2 (2.8)
Certolizumab	1 (1.4)
Canakinumab	1 (1.4)
Vedolizumab	1 (1.4)

Table S2. Location and treatment on diagnosis and on relapse of the 71 patients with an aseptic abscess syndrome

	**Total (*n* = 71)**		
	On diagnosis (*n* = 71)	On relapse (*n* = 44)	On relapse if splenectomy (*n* = 23)
Location of abscesses , *n* (%)			
Spleen	48 (67)	15 (34)	-
Lymph nodes	30 (42)	12 (27)	7 (30)
Skin	17 (24)	8 (18)	3 (13)
Liver	14 (20)	7 (16)	6 (26)
Lung	10 (14)	6 (14)	4 (17)
Muscle	5 (7)	2 (4)	1 (4)
Genitalia (vagina, prostate, testicles)	3 (4)	2 (4)	0
ENT	3 (4)	2 (4)	1 (4)
Kidney	2 (3)	5 (11)	1 (4)
Brain	0 (0)	4 (9)	4 (17)
Pancreas	2 (3)	2 (4)	1 (4)
Breast	1 (1)	1 (2)	0
Treatment, *n* (%)			
Corticosteroids	57 (80)	42 (95)	21 (91)
Colchicine	20 (28)	27 (61)	10 (43)
Immunosuppressants/immunomodulators	16 (22)	30 (68)	14 (61)
Biologics	3 (4)	23 (52)	6 (26)

Table S3. Characteristics of the 71 patients with an aseptic abscess syndrome according to the association or not with an inflammatory bowel disease

	**IBD (*n* = 30)**	**No IBD (*n* = 41)**	***p*-value**
Age (years), mean±SD	26.9±1.77	40.1±3.02	0.0004
Female gender, n (%)	15 (50.0)	19 (46.3)	0.761
Time (months) between 1st symptoms and diagnosis, mean±SD	12.4±4.79	16.1±23.7	0.19
First symptoms, *n* (%)			
Fever	27 (90.0)	34 (82.9)	0.75
Abdominal pain	23 (58.9)	23 (79.3)	0.076
Number of organs involved, mean±SD	2.6±1.8	2.5±1.5	0.88
Main abscess location, *n* (%)			
Spleen	23 (76.6)	28 (68.2)	0.43
Lymph node	16 (53.3)	20 (48.7)	0.70
Skin	10 (33.3)	11 (26.8)	0.55
Liver	7 (23.3)	13 (31.7)	0.43
Lung	6 (20.0)	10 (24.3)	0.66
Associated condition, *n* (%)			
Pyoderma gangrenosum	3 (10.0)	7 (17.5)	0.37
Relapsing polychondritis	0	6 (14.6)	0.02
Spondyloarthritis	1 (3.3)	2 (4.8)	0.74
Laboratory abnormalities			
Anemia	14 (46.6)	15 (36.5)	0.39
Polymorphonuclear neutrophil hyperleukocytosis	27 (90.0)	34 (82.9)	0.39
Increased CRP	28 (9.3)	38 (92.6)	0.91
Liver function test abnormalities	8 (26.6)	10 (24.3)	0.82
Colonoscopy on diagnosis, *n* (%)	14 (46.6)	16 (40.0)	0.57
Splenectomy, *n* (%)	10 (33.3)	13 (31.7)	0.88
Treatment, *n* (%)			
Antibiotics	24 (80.0)	35 (85.0)	0.55
Anti-tuberculosis drugs	5 (16.6)	6 (14.6)	0.81
Corticosteroids	23 (76.6)	34 (82.9)	0.51
Immunosuppressants on diagnosis	8 (26.6)	8 (19.5)	0.47
Immunosuppressants or immunomodulators	19 (63.3)	25 (60.9)	0.84
Biologics	7 (23.0)	16 (39.0)	0.16
Relapse, *n* (%)			
Mean number ± SD	1.3	1.6	0.46
Location of relapse vs diagnosis			
In the same organ, *n* (%)	13 (43.0)	20 (48.7)	0.64

CRP, C-reactive protein; IBD, inflammatory bowel disease; PG, pyoderma gangrenosum; RP, relapsing polychondritis; SD, standard deviation; SPA spondyloarthritis.
